# Designing Health Apps to Support Dietetic Professional Practice and Their Patients: Qualitative Results From an International Survey

**DOI:** 10.2196/mhealth.6945

**Published:** 2017-03-31

**Authors:** Juliana Chen, Jessica Lieffers, Adrian Bauman, Rhona Hanning, Margaret Allman-Farinelli

**Affiliations:** ^1^ School of Life and Environmental Sciences and Charles Perkins Centre The University of Sydney Camperdown Australia; ^2^ School of Public Health and Health Systems University of Waterloo Waterloo, ON Canada; ^3^ School of Public Health and Charles Perkins Centre The University of Sydney Camperdown Australia

**Keywords:** dietetics, apps, app design, mHealth, smartphone

## Abstract

**Background:**

Dietitians are engaging with mobile health (mHealth) technologies, particularly with diet and nutrition apps in their patient care. Despite the plethora of apps available, the majority are not designed with a dietitian’s input.

**Objective:**

The aim of this study was to identify the user preferences of dietitians in relation to tools, resources, and design features for smartphone health apps that would support their dietetic professional practice and their patients.

**Methods:**

As part of a larger international Web-based survey of health-app use among dietitians, three open-ended responses were included for specific exploration of app design features and additional resources or tools that could guide the development of apps for use in dietetic practice and patient care. Inductive thematic analysis of responses was conducted using the qualitative data analysis program, NVivo version 11 (QSR International Pty Ltd), to understand the design preferences and features valued by dietitians.

**Results:**

The responses from 381 dietitian respondents were analyzed. Five key themes were identified. Dietitians wanted access to credible apps, suggesting that dietetic associations should have greater involvement in reviewing and endorsing evidence-based apps for use in dietary counseling. Improvements to the usability of apps, relating to their ease of use and design, were also raised, as self-monitoring of dietary behaviors using existing nutrition apps was deemed to be burdensome. Furthermore, apps providing dietitian-oriented support were favored, for example, those with the ability to streamline the dietary assessment process, so that dietitians could spend more time on dietary counseling and negotiating patient goals for dietary and lifestyle behavior change. Provision of patient-oriented support, such as functionality to tailor apps to patient-specific needs, was also considered important. Finally, respondents valued apps that could integrate into their work systems to enhance the quality of the dietitian-patient relationship.

**Conclusions:**

App developers should draw upon the features and characteristics valued by dietitians to guide their development of apps that support dietetic practice and enhance patient care. Moreover, to achieve better dietitian and patient-centered app design, it is imperative that app developers take a collaborative approach with dietitians, their professional associations, and their patients.

## Introduction

Smartphone ownership among richer, developed countries is nearing ubiquity. According to the Pew Research Center, a median of 68% of adults in advanced economies reported owning a smartphone in 2015 [1] and 77% of smartphone owners had downloaded smartphone apps [2]. Alongside the digital age, the prevalence of obesity and its associated noncommunicable diseases has rapidly increased [3-5], leading to rising social, health care, and economic costs [6]. Capitalizing on the ubiquitous and accessible nature of smartphones and their associated apps, mobile health (mHealth) strategies have the potential to provide cost-effective and scalable health care solutions to manage the escalating burden of disease. In 2015, there were over 160,000 mHealth apps available in the major app stores (eg, Google Play and Apple App store) [7]; approximately two-thirds of which targeted consumer health and wellness, comprising diet and nutrition, fitness, and other lifestyle and stress apps [8]. However, industry reports indicate that the majority of the 45,000 mHealth publishers had information technology backgrounds [7]. Even when publishers included additional team members with medical competencies, often these members were not sourced from the traditional health care industry [7].

Dietitians are trained and skilled experts in diet and nutrition [9] and have recognized roles in delivering effective lifestyle interventions for weight management through the counseling of health behaviors [10,11]. Their expertise could provide app developers and mHealth publishers with valuable insight into best practice treatment strategies to be incorporated into diet and nutrition apps. Within the literature, some studies have documented the input of dietitians in the development of apps intended for use by the public, such as the weight loss app “My Meal Mate” [12] and the gestational weight monitoring app “Eating4Two” [13]. There have also been a number of apps designed by dietitians for use in research but have not yet been implemented for general usage by dietitians [14-17]. Furthermore, since dietitians are using smartphone health apps and other mHealth technologies in patient care [18-20], understanding their experiences may enhance the ongoing development of apps to support the needs of dietetic practice.

There is a paucity of research that has investigated the design features and characteristics that dietitians seek for inclusion in health apps to support their professional practice. One previous study of Canadian dietitians examined factors affecting app use and recommendation in practice. Factors that were found to affect app use and recommendation included those relating to mobile devices and apps, the person and workplace; however, these findings were more centered on the barriers to app use rather than specific app design recommendations [18]. Dietitians and consumers were consulted during the development of a health platform designed for weight management, MyPace [21]; however, feedback was more relevant to the specific design features of the platform. The app design preferences of mobile phone users more generally, have been more commonly explored. Attractive user interfaces, structure, ease of use, personalized features, and accessibility were valued in weight loss apps [22], and usability, cost, and content quality were valued among wellness apps [23]. Among physical activity apps, some features desirable to users included automatic tracking and monitoring of progress toward physical activity goals and an integrated music feature [24].

We have previously determined from an international survey that 62% of dietitians used health apps as an information resource and for patient self-monitoring [19]. In particular, MyFitnessPal and the Monash University Low FODMAP Diet apps were the most commonly recommended by dietitians [19]. This study reports on the qualitative findings from the larger international survey and specifically aimed to identify dietitians’ user preferences regarding the tools, resources, and design features to be included in smartphone health apps that would support their dietetic practice and their patients.

## Methods

### Participants

This study was conducted with dietitians from the United Kingdom, Australia, and New Zealand. The dietetic associations for each respective country assisted in recruitment by distributing a link to the Web-based survey via their weekly member electronic newsletters, social media (Facebook) post, or emails directly to each of its members. Eligible participants had to be a Registered Dietitian (United Kingdom, New Zealand) or Accredited Practising Dietitian (Australia). The recruitment process has been described in more detail elsewhere [19]. Approval for this study was granted by the University Human Research Ethics Committee (approval number: 2015/701).

### Data Collection

An interpretive paradigm was adopted by this study, which sought to understand the individual experience and the meaning they attribute to their actions [25]. As such, open-ended questions were selected as the research method to elicit new and more diversified information, especially on topics or experiences where there is limited information [26]. Open-ended questions also provide opportunities for respondents to share more rich and detailed opinions than that which could be achieved with close-ended questions alone [26,27]. Therefore, as part of a larger cross-sectional survey aiming to investigate dietitians’ use of smartphone health apps and other mHealth technologies in practice, the 3 open-ended questions shown in Textbox 1 were included to allow more specific exploration of app design features and additional resources or tools which could enable health apps to better support dietetic practice. Detailed methods about the piloting and development of the survey have been described elsewhere [19,28].

**Open-ended questions included on health-app design features that would support dietetic practice.**

Questions

What’s your ideal for how health apps for smart devices could be designed and can evolve to be most effective in your dietetic practice?

What additional information, education, resources, or tools could be integrated into apps to help you in your dietetic practice?

Do you have any other advice on what you see as features of a good app, for our teams working on developing apps for smart devices relevant to dietetic practice?

### Data Analyses

Inductive thematic analysis, guided by the framework described by Braun and Clarke [29], was applied to the survey questions. This involved 6 phases: (1) familiarization with the data, (2) initial code generation, (3) searching for themes, (4) reviewing themes, (5) defining and naming of themes, and (6) writing up of results. Coding of the responses was performed using the NVivo qualitative data analysis software version 11 (QSR International Pty Ltd). One researcher (JC) conducted the analysis, coding all the responses to allow for data immersion and to obtain an overall sense of the entire dataset. Generated codes and subsequent themes were checked through a process of ongoing discussion with a second researcher (MAF) who was familiar with the data, before finalization.

## Results

A total of 385 respondents attempted at least one of the 3 open-ended questions included in the larger survey (Q1 n=354; Q2 n=291; Q3 n=234). An additional 185 respondents completed the quantitative study but did not attempt any open-ended questions [19]. Four responses were excluded as they were nonattempts to the questions (eg, ?, -, a, test), thus responses from 381 respondents were analyzed. Table 1 reports the respondent characteristics. Respondents were mainly female (94.8%; 361/381) and aged between 26 and 35 years (41.7%; 159/381). The majority of respondents used health apps in patient care (62.7%; 239/381) and recommended apps to their patients (84.5%; 322/381).

**Table 1:** Respondent characteristics profile (n=381).

Characteristics		n (%)
**Country of dietetic membership**		
	United Kingdom	155 (40.7)
	Australia	213 (55.9)
	New Zealand	13 (3.4)
**Gender**		
	Female	361 (94.8)
	Male	20 (5.2)
**Age (years)**		
	18-25	35 (9.2)
	26-35	159 (41.7)
	36-45	95 (24.9)
	>46	92 (24.1)
**Years in practice (years)**		
	<1	28 (7.3)
	1-5	102 (26.8)
	5-10	77 (20.2)
	10-20	99 (26.0)
	>20	75 (19.7)
**Setting of dietetic practice^a^**		
	Hospital: Inpatient	153 (40.2)
	Hospital: Outpatient	144 (37.8)
	Private Practice	111 (29.1)
	Community	107 (28.1)
	Government and nongovernment organizations for public health	49 (12.9)
	Other^b^	82 (21.5)
**Areas of nutrition management^a^**		
	Weight management	251 (65.9)
	Diabetes	226 (59.3)
	Gastroenterology	139 (36.5)
	Nutrition support	115 (30.2)
	Allergy and intolerances	97 (25.5)
	Cardiology	95 (24.9)
	Geriatrics	92 (24.1)
	Pediatrics	80 (21.0)
	Oncology	68 (17.8)
	Mental health	51 (13.4)
	Renal	43 (11.3)
	Pregnancy/breast feeding	39 (10.2)
	Other^c^	70 (18.4)
**Use of health apps in patient care^d^**		
	Yes	239 (62.7)
	No	142 (37.3)
**Recommend apps to patients**		
	Yes	322 (84.5)
	No	59 (15.5)

^a^Respondents were able to make multiple selections for these questions.

^b^Other categories includes responses with less than 10%: research/academia 7%, sports nutrition 4%, corporate 4%, food service management 4%, indigenous health 4%, and food industry 1%.

^c^Other categories includes responses with less than 10%: sport nutrition 9%, neurology/neurosciences 8%, and eating disorder 2%.

^d^Use of health apps in patient care is defined as dietitians using apps for specific purposes in the nutrition care process (eg, as an information resource, for patient self-monitoring, extra support for patients, dietary assessment tool), and extends beyond recommending apps for patients to use in their own self-management of health.

Thematic analyses of the 3 open-ended questions identified 5 major themes: credibility, usability, dietitian-oriented support, patient-oriented support, and integration into dietitian work systems, most with 2 or 3 associated subthemes. The key findings derived from each theme are discussed. Quotes that are representative of the overall sample have been cited, and where exceptions arose, those responses have also been presented.

### Credibility

This theme captures dietitians’ uncertainty over the credibility of apps, making it difficult for them to recommend apps to their patients. Greater reviewing and endorsement of credible evidence-based apps by dietetic associations and collaboration between app developers and dietitians could improve the confidence of the profession in using and recommending apps in dietetic practice.

#### Reviewing and Endorsement of Apps by Dietetic Associations

Respondents wanted health apps to be reliable sources of up-to-date evidence-based information and to also have scientific evidence backing their efficacy. However, concerns over the accuracy and validity of these apps produced considerable hesitation among respondents when they were considering whether to recommend health apps to their patients. This was particularly the case if apps were to be used as a standalone tool without the support or guidance of a health professional.

However the accuracy of most apps is uncertain and so we always recommend patients use them with caution and in conjunction with the information they receive from us.

r295

Some respondents were overwhelmed by the number and range of health apps available and expressed difficulty in remaining up-to-date with those that were most relevant to their practice and credible to recommend to patients. It was suggested that dietitian professional bodies such as dietetic associations should review these apps and endorse those considered to be credible and safe to their members.

As a registered dietitian we are also not able to promote one product above another as per our code of conduct. It would be nice to have a product which has been reviewed to be accurate and endorsed by a professional body such as the BDA [British Dietetic Association] to enable more active promotion among patients.

r293

Respondents also suggested increased promotion and advertising of the best apps to recommend, such as through distribution of dietetic approved lists of credible apps. Continuing professional development activities, including training workshops, seminars, or webinars could also enhance the profession’s knowledge about the functions and features of particular apps.

#### App Developers to Collaborate With Dietitians

To design credible apps specific to the needs of dietetic practice, respondents proposed greater involvement of dietitians in the app development process. Apps designed in collaboration with dietitians or with dietetic associations were considered to be more acceptable and trusted by the profession.

I have more faith in apps designed by dietitians for use by dietitians!

r287

To have DAA (Dietitians Association of Australia) designed apps - at least the apps are designed by an accredited association.

r209

### Usability

This theme explores the usability of health apps and the app design features which could enhance their ease of use across a range of users, both for the dietitian and their patients.

#### Easy to Use

Improvements in app functionality to make them more straightforward and easy to use was prioritized by respondents, especially because more complex apps could cause confusion to patients and detract from their use. With the time constraints of consultations, respondents also affirmed that apps had to be easy to download and set up.

Easy to use interface, not too complicated. One with minimal set up time (not one million questions about your health to begin).

r46

In describing how food logging and inputting data into an app could be a tedious process for patients, respondents suggested that features such as the ability to duplicate frequently consumed meals and save favorite foods could be more readily incorporated. Tools such as barcode scanners and voice-activated data logging could also make the logging process quicker, easier, and simpler. Photo logging of meals was also proposed as a less-burdensome alternative method to manual food logging in apps for patients. This method would be further assisted with inclusion of other more advanced technologies, such as image recognition to determine portion sizes or nutrient content of foods.

#### Usability for All

Respondents mentioned how apps should provide greater accommodation for a range of user demographics, including different ages, literacy levels, and familiarity with technology among patients. Compatibility across different platforms, including both iOS and Android and across a range of smart devices (eg, smartphones, tablets, older phones) was also specified, since lack of compatibility was a barrier to patient use of certain apps recommended by dietitians.

Equally available on both iOS and Android platforms - many of my younger patients have Android and not all apps are supported on this platform.

r244

It was also commented that apps that should work offline or on little data, particularly to support dietitians servicing patients in remote or rural communities. Respondents also encouraged developers to make apps available for free or at a low cost, citing that paid apps inhibited app uptake by patients.

#### App Design

Simple, user-friendly app designs with easy to read fonts, and basic layout formats that were still visually appealing were sought after by respondents. Respondents wanted textual information and jargon to be minimized, opting for greater inclusion of visuals as a medium for communicating information to patients. App developers were also recommended to create “all-in-one” apps that could carry out multiple activities, citing greater convenience for both the dietitian and their patients.

It would be useful to have an app that had a number of functions - food diary, calorie counter, goal setting and physical activity tracker. It would be easier to recommend one app than three or four to a patient.

r132

However, others preferred to have separate apps that were specific to the nutritional management requirements of their patients.

Less is often more. Don’t try to create an app that can do everything. Have one based on weight loss, a different version for allergies/intolerances etc.

r146

### Dietitian-Oriented Support

This theme describes the app design considerations which should be addressed with regard to dietary assessment and behavior change and the dietitian-specific tools which should be implemented to support dietitian-oriented tasks.

#### Dietary Assessment

Respondents recognized the potential of food diary apps to make the dietary assessment process more streamlined through access to computed and analyzed dietary information from app food diaries, thus allowing more time to be spent on discussing strategies with the patient.

Patient enters their dietary intake, a full nutritional analysis can be done by a program with results e-mailed to the dietitian – reduces time of collecting and analysis dietary info.

r198

However, there was dissatisfaction with the current state of food diary apps. To ensure apps accurately reflected the nutrient composition of the local food supply, respondents emphasized that food databases had to be country specific, rather than being primarily derived from the US-based foods.

More UK relevant apps as a lot of apps tend to be USA-centric and foods in these apps are USA-based which then means patient has to find most relevant food which may be way off UK kcals.

r376

Dietitians also sought after food-based apps, rather than nutrient-based apps, as this would be more complementary to the dietary counseling advice provided by dietitians. Apps that tracked adherence to dietary guideline recommendations or food groups, as opposed to solely focusing on energy and nutrients were suggested.

Those that take food groups into account as opposed to macro- or micro-nutrients, eg, for 1 day there are 3 boxes to tick off, 1 box = a serve of milk/ milk product, 5 boxes for veg etc.

r192

Respondents also highlighted that photo functionalities within apps could enhance the dietary assessment process, especially around the estimation and discussion of appropriate portion sizes.

It would be great if they could involve pictures of the meal so that I can assess portions. Quite often patients underestimate portions... A visual diary can be a very powerful tool even without kJ information.

r162

#### Behavior Change

Health apps were predominantly reported as tools to promote patient self-monitoring, although mainly of weight, diet, and exercise. Respondents wanted greater flexibility in the outcomes tracked, such that personalized and specific goals negotiated with the dietitian could be entered into the app for patients to monitor. Respondents also communicated a desire for the functionality of health apps to extend beyond mere tracking of health behaviors, suggesting that a broader array of automated in-app feedback and encouragement based on patient performance be included to facilitate behavior change.

Offer suggestions when things aren’t going well and encouragement when things are, eg, recognizes a goal has/has not been met.

r340

Others described how health apps could provide extra support and motivation between consultations. Implementation of push notifications or motivational messages derived from the app could also provide reminders, prompt practice, and action to use the app to achieve goals.

#### Dietitian-Specific Tools

Calculators for assisting with anthropometric assessment (eg, body mass index) and estimating energy and nutrient requirements were viewed as valuable tools for dietetic practice. More specifically among respondents working in inpatient hospital settings, there was demand for an app that could calculate and formulate enteral nutrition treatment plans.

They could have the complete compendium of all nutritional feeds and used to work out enteral provision based on calculated requirements inputted, ie, fully functional platform for calculating nutrition needs with stress factors and activity and then work out the different ways of meeting those requirements with feeds.

r254

Respondents also wanted apps to contain or link into practice guidelines, handbooks, and evidence-based information, such as Practice-based Evidence in Nutrition (PEN).

### Patient-Oriented Support

This theme identifies two main strategies for improving patient-oriented support, namely, through the option for tailoring apps to individual needs and by providing patient-specific tools for self-management of health.

#### Tailored to Patient

Respondents considered that the best apps would be modifiable to suit their own dietetic practice and could be customized to adjust for individual patient preferences. Mostly, respondents wanted personalization to occur within the app and not just only in the settings. For example, apps that could enable patient-negotiated tailored goals to be entered and subsequently tracked were valued by respondents. Others suggested using virtual technologies as a creative method for engaging patients on a personal basis.

The ability to personalize in some way - use of an avatar, background design, etc. (to create some 'feel' for the app, and patient buy-in).

r302

As dietitians often provide counseling over a range of nutrition management areas, respondents desired apps that would support a range of their patient’s conditions (eg, cardiovascular disease, coeliac disease, diabetes, obesity). Respondents also highlighted a gap in the apps that were available for renal patients. They suggested that apps tailored to this patient group should provide analysis of key nutrients, including sodium, potassium, phosphate, and fluids, to allow for the monitoring or management of kidney disease.

A good start would be a very simple app for adhering to a fluid restriction. Another good option would be a traffic light system for potassium and phosphate foods, an app for sodium restriction too would be good.

r218

#### Patient-Specific Tools

To increase knowledge and empower patients to self-manage their health, respondents wanted their patients to be able to access to educational resources on different health conditions and nutritional recommendations directly from within the app and for the app to also link to other internet resources. Built-in videos or podcasts in the app were also suggested as a more engaging format to explain diet-disease relationships to patients.

It was also suggested that apps could provide meal or menu plans with attached recipes. This information could then be used to generate shopping lists for general healthy eating and specific diets. Tools that could help patients to make choices about healthier food alternatives, particularly for snack options, or to determine whether a food was appropriate to special dietary needs, were also perceived to be helpful for patients.

An app for helping food allergic patients choose safe packaged foods, which is regularly updated as products frequently change.

r8

Scan grocery items and it flashes Red, Amber or Green depending on the programed nutrient to include or exclude from the diet.

r19

### Integration Into a Dietitian Work Systems

This theme highlights how sharing of health-app data could improve patient-provider communication and care through enabling greater integration of these mHealth technologies into dietitian work systems.

#### Sharing of App Data

To improve workflow, respondents commented that patient health-app data should be sharable or exportable from apps for direct viewing in dietitian work systems, citing that reviewing app records on a patient’s phone was impractical. Respondents suggested several ways to share this data including email, Bluetooth synchronization, record printouts, and the ability to upload records onto a website or platform. However, ensuring the security and privacy of these app records was emphasized. The efficiency of work processes could also be improved by app data linkage to electronic health records.

Direct links to patient's electronic health records for information exchange and capture of information as part of their health records. The ability for patients to get their results, see the goals we've agreed in consultations.

r249

Dietitians also felt that having access to patient health-app data would improve patient-provider communication, as well as providing them with more opportunities to provide real-time feedback and support between consultations based on their patient’s monitoring.

Food diary which allows access remotely to patient information so that it can be analyzed before clinic or as concerns come up.

r279

## Discussion

### Principal Findings

To our knowledge, this study is one of the first to identify design considerations, including features and tools, for apps supportive of dietetic practice. Dietitians prioritized several design aspects, including the credibility and usability, including ease of use and the design of apps. Apps targeted toward dietitian- and patient-oriented support and that could integrate into dietitian work systems were also regarded favorably. These findings provide guidance to app developers about the fundamental characteristics to address while designing dietitian- and patient-oriented apps.

Dietitians are guided by codes of professional conduct to provide evidence-based practice, which extends also to the promotion of products, including health apps [30-32]. However, recommending apps in a professionally responsible way has been challenging since regulation only exists for apps considered to be medical devices [33-35], and health and wellness apps are left largely ungoverned. Furthermore, there are growing concerns over the credibility and evidence base of a range of mHealth apps that may be recommended in dietetic practice, such as weight management [36-41], diabetes [42-44], and physical activity [45,46] apps. Some studies have attributed the poor credibility of these apps to low health care professional involvement in app development [38,41]. Health care expert involvement in medical urology app development has been found to positively influence app downloads, suggesting that collaboration with health care specialists gives users greater assurance of the safety and credibility of an app [47]. As such, coinciding with our previous recommendations formulated on the basis of the COM-B model [19], involvement of dietitians and dietetic associations in the development as well as reviewing and subsequent endorsement of credible and reputable apps are necessary to enhance the confidence of the profession and their patients in recommending and using apps, respectively. Dietetic associations, such as Dietitians of Canada [48] and the US Academy of Nutrition and Dietetics [49] have headed the development and reviewing of credible apps for patient use and to support the dietetic profession.

According to the Technology Acceptance Model, perceived usefulness and perceived ease-of-use of a technology predicts users’ attitudes and behavioral intentions toward accepting the technology, thus affecting subsequent technology use [50]. Usability-related characteristics, such as ease-of-use, were not only valued among our responding dietitians but also were a positive contributor to users’ ratings within app stores [51] and were valued by mobile phone users in relation to wellness [23] and popular diet and weight loss apps [22,52]. An app requiring low effort to use is an imperative design consideration since both commercial and researcher-designed health apps typically experience a rapid decline in app use over time and low long-term retention [53-55]. Usability testing of a popular dietary app, MyFitnessPal, revealed users’ dissatisfactions over inconveniences in food logging and complex structure which resulted in a loss of interest in using the app [56]. Tools such as barcode scanners and image-based food logging could minimize user burden and allow patients to maintain compliance with tracking, while still providing valid measures of intake [57-59]. Furthermore, to improve adoption of apps, attractive user interfaces and simple to navigate designs should be included. In Web patient portal use, Web aesthetic simplicity (ie, cohesiveness, structure, and easiness to understand) was a significant antecedent variable to patients’ acceptance and use of patient portals [60]. App developers also need to engage in more user-testing during the development of apps and incorporate the service user feedback in an iterative design process to produce more dietitian- and patient- oriented apps.

When considering that diet and nutrition apps can automatically calculate energy, macronutrients, and micronutrient values from foods entered, apps present themselves as desirable tools to streamline and support dietitian-led dietary assessment. Yet, app developers should re-evaluate the quantitative approaches to dietary assessment currently implemented within apps, given that few dietitians consider apps as reducing the time for dietary assessment [19] and apps currently appear to lack of effectiveness in improving diet quality [61]. Instead, assessing the overall diet quality and interpreting and translating these dietary patterns into practical and meaningful food-based dietary advice would be more useful to both dietitians and their patients. Echoing conclusions drawn in the literature [36-38,45,62-64], our respondents recommended incorporation of a broader range of behavior change techniques, beyond self-monitoring. Notably, dietitians wanted apps to motivate patients and prompt them to practice health behaviors which could remind and encourage ongoing progress toward goals and encourage behavior change. Inclusion of automated motivational text messages or app push notifications have been found to improve physical activity [65], and when administered as part of a multicomponent mHealth lifestyle intervention, prevented weight gain and improved dietary behaviors [66].

As the health care system shifts away from the delivery of passive care to engaging patients as partners in their own health care, health apps present real-time opportunities to support and empower patients in making positive health behavior choices outside dietetic consultations. However, the absence of tailored goals and feedback is a major shortcoming identified in diet and nutrition apps for weight management [37,38,41]. The ability to input individualized goals within an app, such as those negotiated with dietitians would enable the tracking of more specific health behaviors relevant to the patient. There is only one known app—eaTracker developed by the Dietitians of Canada [67]—that supports the personalization of goals beyond generic pre-set targets of energy intake and weight loss. Additionally, providing personalized nutrition advice via mHealth technologies has been found to significantly improve selected dietary outcomes [68,69] and is an important consideration for developing effective apps. For example, remote and real-time delivery of daily tailored feedback messages significantly reduced energy and saturated fat intake, with changes maintained at 24 months [69]. Use of avatars might also be a method for personalizing the user experience in apps. They have been found to be a highly acceptable medium for modeling weight loss behaviors [70] and may engage and motivate users to change behaviors, such as promoting delayed gratification and dietary regulation [71] through embodying the patient’s ideal self.

Individuals have previously expressed that sharing health-app records with their health professional would be useful to their care [72]. Reports, however, indicate limited sharing of these records with health professionals or dietitians [72,73], possibly attributed to individuals’ perceptions that health professionals had little interest in their health-app records [72]. Contrary to patient beliefs, our responding dietitians wanted access to their patients’ health-app records, particularly to support the dietary assessment process. However, with few commercial mHealth apps having the capability to export user data [8,38], reviewing of patient progress with health apps has often been an infrequent and informal procedure for dietitians consisting of verbal discussion rather than direct viewing of the health-app data [19]. Enabling patients to synchronize, share, or export health-app data into their dietitian’s existing work systems could enhance the two-way communication between dietitians and their patients. The increased connectivity and access to records may create opportunities for dietitians to address patient lapses and deliver more dynamic behavioral strategies to support patient compliance with dietary recommendations. App developers also need to ensure that these functions integrate seamlessly into current practice workflow to avoid imposing addition burdens on time and effort for the dietitian in adopting new systems.

### Limitations

Although some respondents explicitly specified having no additional feedback regarding app design, it is not clear whether respondents who did not complete the open-ended questions had no further comments because they were in fact satisfied with the current state of apps, or whether they did not know what answers to provide and so left a blank response. The cross-sectional nature of this survey also poses the possibility of sampling biases, whereby greater willingness to respond to the open-ended questions may have come from more interested individuals and existing app users. However, the demographic profile of these respondents to the open-ended questions is comparable with that of the larger international survey, which was determined to be representative of the wider dietetic profession [19]. Furthermore, although adequate representation of the perspectives of nonusers is necessary, yet without experience in using existing health apps, the scope of suggestions provided by non–app users may be limited. If app developers perceive that the recommendations put forward regarding app design features already exist, they may be less inclined to develop apps further to support dietitian and patient needs. On the contrary, existing app users are likely to have richer and more feasible recommendations to guide and improve app development and design. It should also be noted that although understanding dietitian and user preferences may allow for more suitable apps to be designed for dietetic practice, this does not necessarily guarantee treatment effectiveness. Therefore, interventions studies are required to confirm which specific design features will provide the most support to dietetic practice and elicit significant effects on patient outcomes.

### Conclusions

This study provides guidance to app developers of the features and characteristics of smartphone health apps valued by dietitians and highlights improvements for the design of health apps. In particular, dietitians asserted that apps should be credible and easy to use in order for them to more effectively support dietetic practice and dietitian’s recommendations of these apps. Greater collaboration between app developers and dietitians or their professional associations were also viewed as critical for achieving dietitian and patient-centered app design and integration into dietitian work systems. However, further investigation is required to determine the app features that offer the most support to dietitians in improving patient health outcomes.

## Abbreviations

mHealth: mobile health
PEN: Practice-based Evidence in Nutrition
